# Transgenerational Effects of Stress Exposure on Offspring Phenotypes in Apomictic Dandelion

**DOI:** 10.1371/journal.pone.0038605

**Published:** 2012-06-18

**Authors:** Koen J.F. Verhoeven, Thomas P. van Gurp

**Affiliations:** Department of Terrestrial Ecology, Netherlands Institute of Ecology, Wageningen, The Netherlands; University of Leeds, United Kingdom

## Abstract

Heritable epigenetic modulation of gene expression is a candidate mechanism to explain parental environmental effects on offspring phenotypes, but current evidence for environment-induced epigenetic changes that persist in offspring generations is scarce. In apomictic dandelions, exposure to various stresses was previously shown to heritably alter DNA methylation patterns. In this study we explore whether these induced changes are accompanied by heritable effects on offspring phenotypes. We observed effects of parental jasmonic acid treatment on offspring specific leaf area and on offspring interaction with a generalist herbivore; and of parental nutrient stress on offspring root-shoot biomass ratio, tissue P-content and leaf morphology. Some of the effects appeared to enhance offspring ability to cope with the same stresses that their parents experienced. Effects differed between apomictic genotypes and were not always consistently observed between different experiments, especially in the case of parental nutrient stress. While this context-dependency of the effects remains to be further clarified, the total set of results provides evidence for the existence of transgenerational effects in apomictic dandelions. Zebularine treatment affected the within-generation response to nutrient stress, pointing at a role of DNA methylation in phenotypic plasticity to nutrient environments. This study shows that stress exposure in apomictic dandelions can cause transgenerational phenotypic effects, in addition to previously demonstrated transgenerational DNA methylation effects.

## Introduction

Plant phenotypic responses to environmental inputs, broadly termed phenotypic plasticity, can have an immediate expression but also a delayed expression within generations or even a transgenerational expression, suggesting mechanisms for sustained memory of environmental experiences. Delayed responses are known for instance in plant-pathogen interactions, where an initial mild attack does not trigger an immediate defense response but prepares the defense machinery for a more rapid and vigorous response upon a second attack in the future [1]. Transgenerational responses, or parental environmental effects, are well-documented in ecological literature and can impact offspring traits such as (timing of) germination, seedling growth characteristics, resource allocation, plant architecture and chemical profiles [2], [3], [4], [5]. Their adaptive value has been demonstrated in natural environments [6]. Parental effects may evolve as adaptations when offspring environmental conditions are predictable from the parental environmental conditions and when they enhance offspring performance under those conditions [7], [8], [9].

The underlying memory mechanisms for delayed and transgenerational stress responses are often unknown, but among a number of candidate mechanisms the stable regulation of gene activity via epigenetic modifications is often proposed [10], [11], [12]. Epigenetic mechanisms may be involved especially when the transgenerational effect is expressed for more than one offspring generation [13], [14], [15]. The involvement of DNA methylation is suggested by observations in *Arabidopsis* that experimental manipulation of DNA methylation using 5-azacytidine affects the expression not only of within-generation phenotypic plasticity [16] but also of transgenerational phenotypic plasticity [17]. Parental effects are also affected in mutants with compromised small RNA-dependent gene silencing machinery [15], [17].

The model of epigenetic regulation of transgenerational phenotypic plasticity assumes that exposure to environmental stress triggers epigenetic changes at specific stress response-related genes which are subsequently stably transmitted through meiosis to offspring; or alternatively that epigenetic marks at these genes are established *de novo* in offspring, directed perhaps by small RNAs that were produced by and inherited from stress-exposed parents [18]. Current evidence for such *detection-based* epigenetic inheritance [19] is limited and the idea remains controversial [20]. However, individual steps in these hypothesized pathways have recently been reported. For instance, artificial herbivory treatment in *Mimulus guttatus* causes increased leaf trichome density in unexposed offspring plants [21] and this is associated with heritably altered expression of a candidate gene involved in trichome production [22]; involvement of DNA methylation or other epigenetic mechanisms has yet to be demonstrated. In *Arabidopsis* abiotic stress exposure in parents affects offspring traits, DNA methylation and global gene expression patterns [17] and recently it was shown that DNA methylation changes at individual genes are correlated to expression of those genes in offspring of salt-stressed plants [23]. In rice, nitrogen deficiency caused DNA methylation changes that were transmitted to some second-generation offspring but not to others, and this variation in epigenetic inheritance was associated with differences in transgenerational phenotypic plasticity in response to nitrogen deficiency [24].

**Figure 1:**
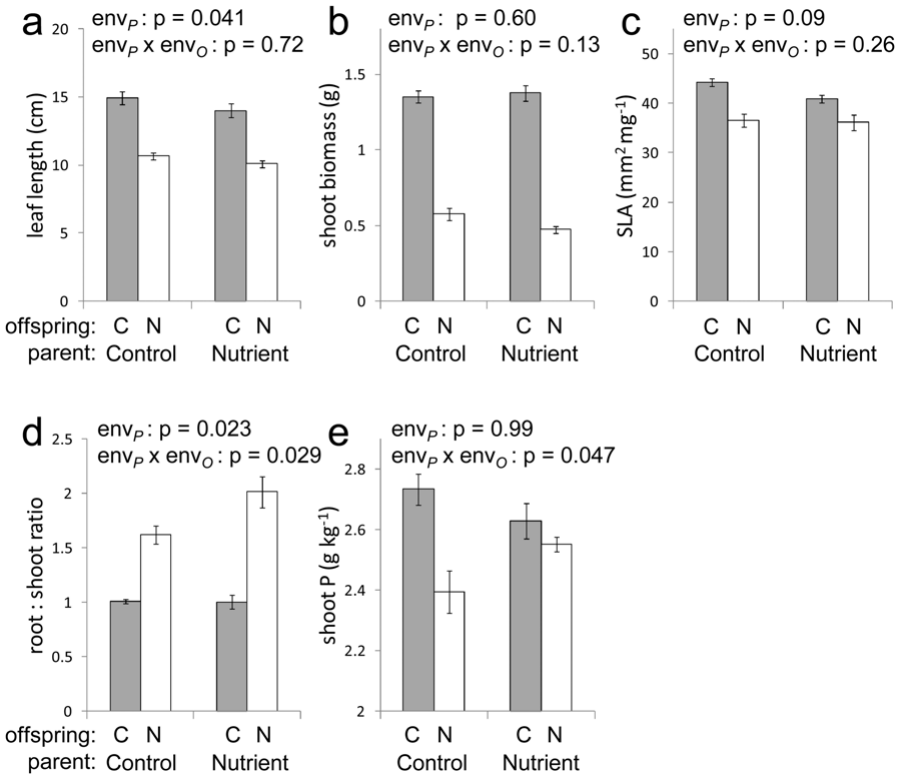
Experiment 1: effect of parental nutrient stress on offspring traits. Leaf length (a); shoot biomass (b); specific leaf area (c); root ∶ shoot ratio (d); and shoot phosphorus content (e), of offspring grown under control conditions (grey bars, offspring environment ‘C’) and nutrient stress (open bars, offspring environment ‘N’) (means ± SEM). P values are shown for tests of parental environment and the parental environment×offspring environment interaction.

**Figure 2:**
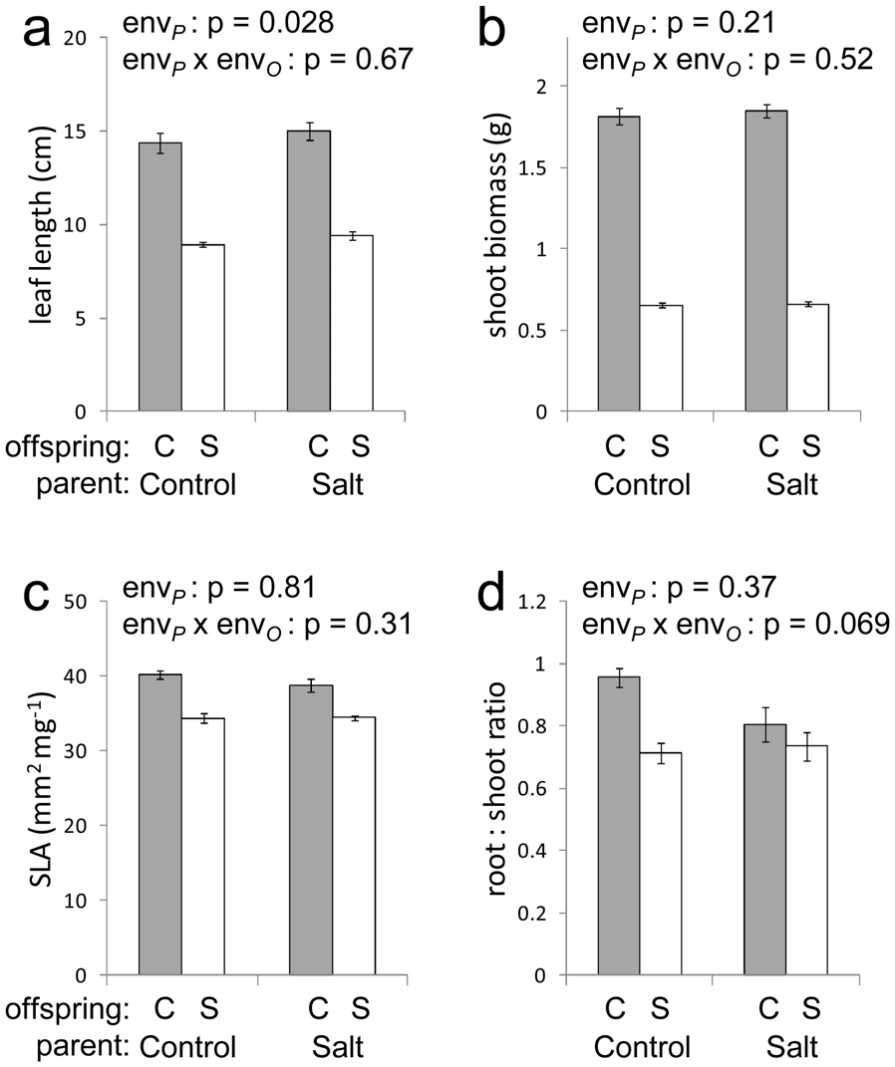
Experiment 1: effect of parental salt stress on offspring traits. Leaf length (a); shoot biomass (b); specific leaf area (c); and root ∶ shoot ratio (d), of offspring plants grown under control conditions (grey bars, offspring environment ‘C’) and high-salt stress (open bars, offspring environment ‘S’) (means ± SEM). P values are shown for tests of parental environment and the parental environment×offspring environment interaction.

To date, environment-induced heritable epigenetic changes have been demonstrated in very few plant species, including apomictic dandelions (*Taraxacum officinale*, common dandelion, Asteraceae). In these plants we previously showed that exposure to environmental stresses enhances the probability of DNA methylation changes at methylation-sensitive AFLP (ms-AFLP) loci; and many of the stress-induced DNA methylation changes are faithfully transmitted to offspring [25]. The dandelion system is convenient for such studies because apomixis (seed production without fertilization, where offspring are thought to be clonal copies of the mother plant) permits the evaluation of epigenetic effects in absence of DNA sequence variation between individuals. However, as pointed out by several authors [26], [27], insight in phenotypic effects that accompany the observed epigenetic modifications in dandelion is required to evaluate the potential relevance of epigenetic inheritance for the species' ecology and microevolution.

Here, we report a series of experiments aimed at evaluating effects of parental stress exposure on offspring phenotypes in apomictic dandelions. Specifically, we address the following questions: (1) Does exposure to the same stresses that trigger heritable DNA methylation changes also cause transgenerational effects on offspring phenotypes? (2) Are transgenerational effects consistent between different apomictic genotypes? (3) Does the expression of transgenerational effects depend on DNA methylation? Parental effects can show stochastic expression and poor repeatability of published effects has been a recent matter of concern [17], [28]. We therefore aimed to gain insight in the robustness of observed parental effects and present the unbiased outcomes of a set of experiments that partly replicate each other.

## Results

### Experiment 1: Parental effects in AS34

Environmental stress treatments significantly affected the weight of seeds produced in the parental generation (mean seed weight 0.853 mg in controls; 0.719 mg under nutrient stress; 0.644 mg under salt stress; 0.916 mg after JA treatment; 0.869 mg after SA treatment; based on 8–48 seeds per group, p<0.001 in one-way ANOVA). After statistically controlling for individual seed weight differences, significant parental effects of JA and SA exposure were detected in offspring specific leaf area (Table 1). Parental effects of nutrient stress were detected in offspring leaf length, root ∶ shoot biomass ratio, and shoot P content (Fig. 1). Parental effects of salt stress were detected in offspring leaf length and, subsignificantly, in root ∶ shoot biomass ratio (Fig. 2). These effects were either expressed as main effects of parental environments or in interaction with offspring environments. Parental nutrient stress caused a reduction in offspring leaf length (Fig. 1a) whereas parental salt stress caused an increase in offspring leaf length (Fig. 2a), irrespective of offspring environments. Offspring of nutrient-stressed plants showed an enhanced root ∶ shoot ratio response when subjected to nutrient stress compared to offspring of control plants but no difference in root ∶ shoot ratio was observed in the control environment (Fig. 1 d). This enhanced root ∶ shoot response was accompanied by a maintaining of higher tissue phosphorus levels under nutrient stress (Fig. 1e).

**Table 1:** Experiment 1: effects of parental JA and SA treatment on traits of offspring raised in common control environments (experiment 1, genotype AS34; means ± SEM).

	JA-experiment			SA-experiment		
	parental environment			parental environment		
trait	control	JA	p value	control	SA	p value
Leaf length (mm)	13.9 (0.5)	13.3 (0.5)	0.557	13.6 (0.6)	14.8 (0.5)	0.206
Shoot biomass (g)	2.68 (0.13)	2.65 (0.09)	0.692	2.84 (0.05)	2.88 (0.07)	0.778
SLA (mm^2^ mg^−1^)	35.4 (0.5)	38.0 (0.5)	0.012	35.8 (0.5)	33.7 (0.4)	0.008

**Figure 3:**
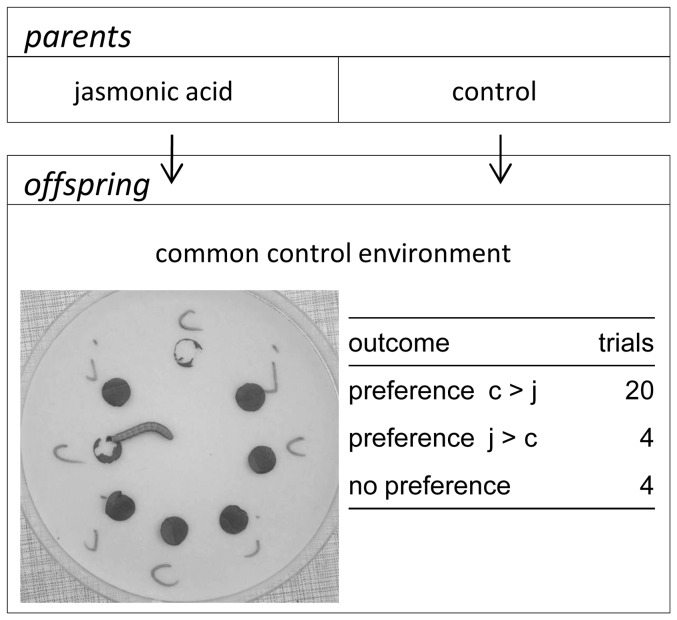
Experiment 1: design and results of *Spodoptera exigua* choice feeding assays. Inset picture: J, offspring of JA-treated plant; C, offspring of control plant. Inset table shows for each independent trial if more tissue was consumed from JA-offspring or from control-offspring leaf discs.

*Spodoptera exigua* larvae preferred to feed on leaf discs from offspring of control plants over offspring of JA-treated plants (Fig. 3). In 16 trials, more leaf tissue was eaten from control-offspring than from JA-offspring and in 4 trials more JA-offspring leaf tissue was eaten (Sign test of equal probabilities: p = 0.031). Four trials were excluded from analysis because no leaf tissue had been consumed after 30 minutes or because equal amounts were consumed from JA-offspring and control-offspring leaf discs.

### Experiment 2: replication in different apomictic genotypes

**Figure 4:**
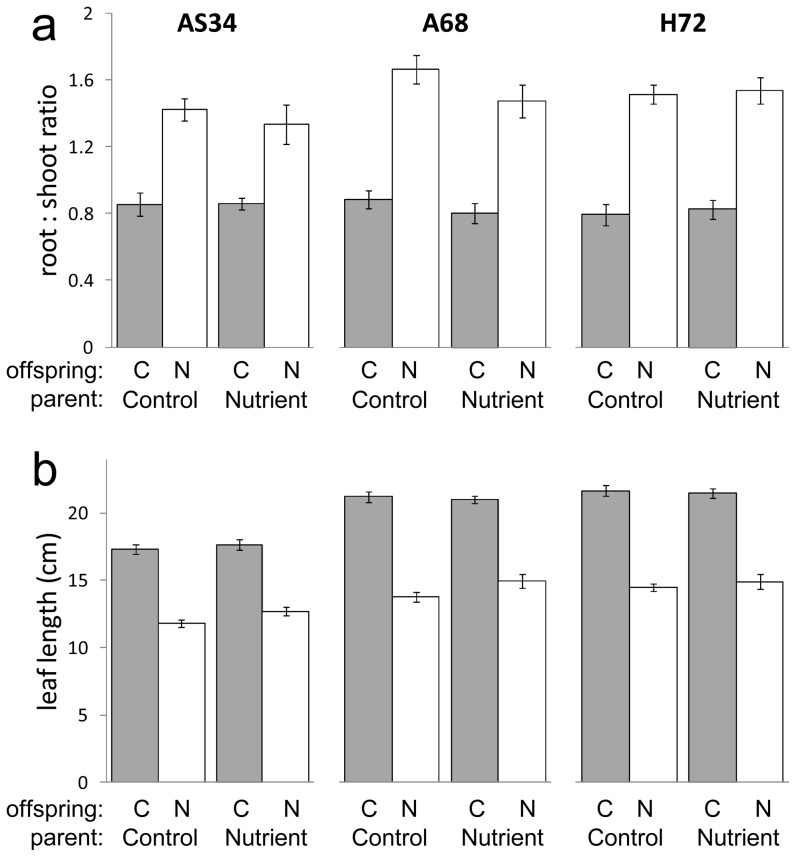
Experiment 2: effect of parental JA-treatment on specific leaf area in three apomictic genotypes. Offspring plants are grown under control conditions (shown are least squares means that are adjusted for block effects ± SEM). Grey bars: offspring of control plants; Open bars: offspring of JA-treated plants. P values are shown for tests of parental JA treatment (JA_P_) and the parental JA treatment×genotype interaction (G×JA_P_).

As in experiment 1, parental effects of JA treatment were absent for leaf length (data not shown) but were visible, albeit subsignificantly, in SLA where different genotypes tended to differ in their expression of the parental effect (Fig. 4). The parental JA-treatment effect in AS34 was similar as in experiment 1 (contrast test of parental JA-effect in AS34: F_1,25_ = 4.0, p = 0.057). The effect on SLA was driven by a parental JA-effect on offspring leaf biomass (p<0.05), not offspring leaf surface area (p>0.05; data not shown). As in experiment 1, *S. exigua* larvae tended to show a preference for leaf tissue from control-offspring over leaf tissue from JA-offspring (Table 2) but this trend was not significant (8 trials versus 4 trials, Sign test of equal probabilities: p>0.05). A preference was absent in the other two genotypes.

**Table 2:** Experiment 2: effects of parental JA treatment on feeding preference by *Spodoptera exigua* larvae in three apomictic genotypes.

trial outcome	AS34	A68	H72
preference C>J	8	10	10
preference J>C	4	11	9
no preference	15	9	8

J, offspring of JA-treated plant; C, offspring of control plant. (see also Fig. 3).

**Figure 5:**
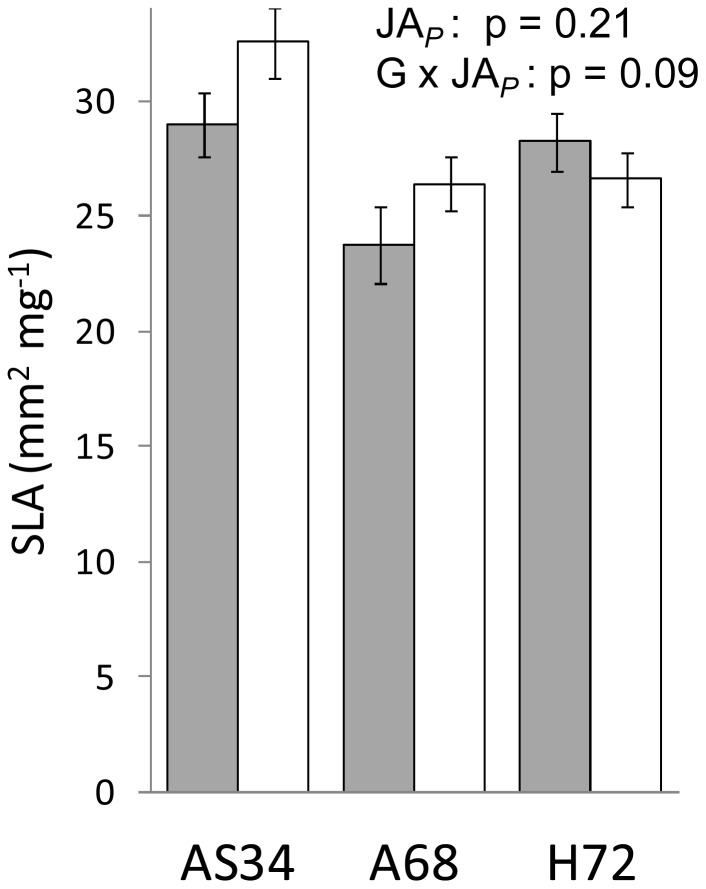
Experiment 2: effect of parental nutrient stress on offspring. Root ∶ shoot biomass ratio (a) and leaf length (b) in three apomictic genotypes, grown under control conditions (grey bars, offspring environment ‘C’) and nutrient stress (open bars, offspring environment ‘N’) (means ± SEM). See Table 2 for p-values.

Parental effects of nutrient stress in experiment 2 showed little consistency with experiment 1. No significant effect of nutrient stress was detected on offspring root ∶ shoot ratio or on the root ∶ shoot response to nutrient stress (Table 3, Fig. 5). Nutrient limitation resulted in reduced leaf length but this reduction was less severe in offspring of nutrient-stressed plants compared to offspring of control plants (significant parental environment×offspring environment interaction, Table 3). This pattern differed qualitatively from the parental main effect on offspring leaf length that was observed in experiment 1 (compare Fig. 5b and Fig. 1a).

**Table 3:** Experiment 2: effects of parental nutrient stress on offspring traits in three apomictic genotypes.

	leaf length		shoot biomass^1^		SLA		root ∶ shoot	
	F	p value	F	p value	F	p value	F	p value
Initial seed weight (1)	1.5	0.23	2.0	0.16	0.2	0.66	0.1	0.78
Replicate block (9)	5.6	<0.001	29.3	<0.001	2.7	<0.01	9.1	<0.001
Block×Nutrient_offsp_ (9)	0.5	0.85	6.9	<0.001	1.2	0.34	1.1	0.41
Block×Genotype (18)	0.6	0.91	2.4	<0.01	1.1	0.37	1.2	0.29
Genotype (2)	68.6	<0.001	34.9	<0.001	1.0	0.39	1.2	0.30
Nutrient_offspring_ (1)	1069	<0.001	4035	<0.001	0.0	0.92	411	<0.001
Genotype×Nutrient_offsp_ (2)	13.6	<0.01	2.7	0.08	0.4	0.70	4.2	0.02
Nutrient_parent_ (1)	0.1	0.70	0.1	0.72	0.5	0.48	2.4	0.13
Nutrient_parent_×Genotype (2)	0.8	0.68	2.9	0.062	0.5	0.59	2.1	0.13
Nutrient_parent_×Nutrient_offsp_ (1)	4.5	0.035	0.8	0.38	0.4	0.54	1.2	0.27
Nutr_parent_×Nutr_offsp_×Gen. (2)	1.2	0.56	1.6	0.22	0.5	0.61	0.2	0.86

1 log-transformed prior to analysis to meet ANOVA requirements.

ANOVA test results are shown for all model factors; factors of special interest are those that include parental nutrient treatment (nutrient_parent_). Error degrees of freedom = 66, model term degrees of freedom are in parentheses.

### Experiment 3: zebularine effects on nutrient stress response

Zebularine treatment decreased plant biomass, leaf length and root ∶ shoot ratio, and increased SLA (data not shown). It also significantly affected responses to nutrient limitation for all traits measured (zebularine concentration×offspring nutrient environment interactions, Table 4). No effects of parental nutrient stress were observed on offspring traits: parental nutrient treatment and all interactions including this term were non-significant in all traits (Table 4).

**Table 4:** Experiment 3: ANOVA test results for effects of nutrient limitation and parental nutrient limitation on offspring traits in AS34, as affected by germination in different zebularine concentrations.

	leaf length		total biomass		SLA		root ∶ shoot	
	F	p value	F	p value	F	p value	F	p value
Replicate block (9)	0.8	0.59	0.8	0.62	0.9	0.55	1.1	0.41
Nutrient_offspring_ (1)	252.5	<0.001	221.8	<0.001	4.4	0.04	267.0	<0.001
Nutrient_parent_ (1)	1.5	0.22	0.2	0.63	0.1	0.83	1.3	0.25
Nutrient_parent_×Nutrient_offsp_ (1)	1.0	0.32	0.2	0.66	0.1	0.72	0.3	0.58
Zebularine (2)	47.0	<0.001	180.4	<0.001	16.1	<0.001	62.3	<0.001
Zebu×Nutrient_offspring_ (2)	5.7	<0.01	80.7	<0.001	2.8	0.07	3.9	0.024
Zebu×Nutrient_parent_ (2)	0.8	0.44	0.8	0.47	0.1	0.89	0.8	0.46
Zebu×Nutr_parent_×Nutr_offsp_ (2)	0.8	0.47	1.3	0.28	0.1	0.93	0.2	0.81

Error degrees of freedom = 110, model term degrees of freedom are in parentheses.

## Discussion

Focusing on the same stress treatments that were previously shown to induce DNA methylation changes [25], this study evaluated transgenerational phenotypic effects in apomictic dandelions. Such phenotypic effects are not necessarily functionally related to previously observed DNA methylation changes at ms-AFLP loci. But demonstrating inherited effects both on DNA methylation and on phenotypes creates a basis for subsequent targeted analysis of transgenerational epigenetic inheritance via heritable DNA methylation modifications at stress response-related genes.

Transgenerational effects were observed in several traits and in response to several stresses. The effects described here are in line with observations in other plant species. For instance, an increased root ∶ shoot ratio in offspring of nutrient-limited parents was previously observed in *Polygonium persicaria* [29]. Nutrient-limited plants are known to invest more in root tissue [30], as expected when plants aim to capture more of a limiting resource [29]. JA-treatment and herbivory were previously shown to increase progeny resistance to caterpillar herbivory in *Raphanus raphanistrum* [31], [32] and in *Arabidopsis* and tomato [15]. Likewise, experimental leaf damage in *Mimulus guttatus* caused increased trichome density in offspring, presumably deterring herbivores [21]. Both the nutrient effect and the JA/herbivory effect appear to enhance offspring abilities to cope with the same stresses that parents were exposed to, suggesting an adaptive significance of transgenerational plasticity. Such potentially adaptive transgenerational effects may be particularly relevant for asexual species, which have limited opportunity to genetically adapt to environmental changes. Enhanced phenotypic plasticity in apomictic dandelions compared to non-apomictic (sexual) conspecifics has been demonstrated previously [33] and can contribute to the ability of apomicts to successfully cope with environmental variation. Extending the scope of plasticity across generations may further enhance this ability.

The transgenerational effects of JA-treatment on specific leaf area and herbivore resistance showed consistent trends in replicated experiments in one genotype (AS34) but these effects were not detected in the other two genotypes. Previous studies have often reported that parental effects are genotype-specific [21], [22], [34], [35], [36]. However, effects of parental nutrient stress were not consistently expressed between replicate experiments even within the same genotype. Especially given this limited consistency it needs to be considered to what extent significant results in our study are statistical artifacts or type 1 errors, which occur at a rate of 5% in absence of a true effect. Across all experiments presented in this study we performed 61 statistical tests of parental effects (either as main effect or in interaction with other model terms). Ten of these were significant (p<0.05), whereas only three (5%) are expected to occur due to chance. Thus, the total set of results provides evidence for the existence of parental effects in apomictic dandelions, but due to inconsistent expression some of the individual effects (especially those related to nutrient stress) can only be tentatively interpreted. Variable effects of nutrient stress were recently reported also in rice, where heritable effects of nutrient stress on DNA methylation and offspring stress tolerance were transmitted to some offspring individuals but not to others within the same experiment [24].

Inconsistent expression and limited reproducibility of transgenerational phenotypic effects is a topic of recent debate in plant epigenetics. For instance, transgenerational effects of UV-C and flagellin exposure in *Arabidopsis* on somatic recombination rate were observed to persist for multiple unexposed offspring generations in one study [37] but these results proved difficult to replicate by others [17], [28]. Parental environmental effects are often genotype-specific [21], [22], [34], [35], [36], their induction in the parental generation and their expression in the offspring generation may be sensitive to specific conditions such as the severity of stress [38], and expression of effects might decrease with seed age [39]. Clearly, better insight in the specific conditions under which parental effects are induced and expressed is necessary to evaluate the scope of these effects. It is conceivable that the developmental stage of the parent plant during stress exposure is critical, or that subtle differences between replicated experiments override parental effects. For instance, in one study a heritable effect of herbivory treatment on offspring insect resistance was observed in several but not in all replicated experiments [15].

Experimental evidence for a role of DNA methylation in transgenerational phenotypic effects can come from experimental manipulation of DNA methylation. Exposing germinating *Arabidopsis* seeds to the demethylation agent 5-azacytidine affected the expression of phenotypic plasticity in response to nutrient environments [16] and also the expression of transgenerational phenotypic plasticity in response to parental salt environments [17]. Although demethylation agents such as 5-azacytidine may have additional effects on genome integrity besides reducing methylation levels, such observations are suggestive of a role of DNA methylation in (transgenerational) phenotypic plasticity. Using a similar experimental approach we showed that the within-generation response to nutrient stress was affected by zebularine in all traits measured (leaf length, plant biomass, specific leaf area and root ∶ shoot ratio). This suggests a role of DNA methylation in the normal plant response to nutrient limitation, consistent with results in *Arabidopsis* [16]. However, this experiment failed to show evidence for a transgenerational nutrient effect on offspring traits. It could therefore not be determined whether the expression of transgenerational effects is affected by experimental demethylation.

Epigenetic inheritance, for instance via stress-induced DNA methylation modifications, is only one of several candidate mechanisms to explain heritable effects of environments on offspring phenotypes. Many transgenerational stress experiments, including ours, evaluate effects only in first-generation offspring of stressed plants. To distinguish epigenetic from other possible mechanisms (such as maternal transmission to the developing embryo of hormones, non-nuclear genes, enzymes or toxins) it will be important in subsequent studies to evaluate the persistence of induced effects also in second-generation offspring [20]. Environment-induced effects that persist for several offspring generations in absence of the inducing trigger can be explained by stable epigenetic changes but are more difficult to explain by many of the other maternal effect mechanisms. Multigenerational persistence of stress-induced phenotypes has been documented in plants [14] but to date such reports are quite scarce.

In conclusion, this study provides support for the existence of parental environmental effects in apomictic dandelions. It also highlights stochasticity in the expression of these effects, in agreement with recent reports on transgenerational effects in plants. The ecological relevance and scope of these effects might be questioned if their expression is inconsistent or sensitive to very specific conditions. However, a better understanding of the factors responsible for variation in expression is necessary in order to evaluate the ecological relevance of the effects. For instance, mild levels of parental stress, which may be ecologically realistic, can trigger stronger parental effects than severe stress that may be typical of lab experiments [38]. The observations that stress exposure in apomictic dandelions leads to heritably modified DNA methylation [25] and modified offspring phenotypes (this study) may hint at the relevance of epigenetic mechanisms for generating heritable variation and transgenerational plasticity within the genetically uniform lineages of this species, which can contribute to their adaptive potential.

## Materials and Methods

### Ethics statement

No specific permits were required for the described studies.

### Plant material and growing conditions

Asexual variants of the common dandelion, *T. officinale*, are polyploid (usually triploid, 3x = 24) obligate apomicts that produce clonal seeds in a process that involves unreduced egg cell formation (diplospory), parthenogenic embryo development and autonomous endosperm formation [40]. We used progeny from three apomictic genotypes: AS34, which was produced in an experimental cross between a sexual diploid mother and diploid pollen from a triploid father [41]; and A68 and H72, which are both natural apomicts that were previously collected from the field. All genotypes had been propagated in the greenhouse for at least one generation prior to experimentation. Unless stated otherwise for individual experiments, seeds were germinated on water-saturated filter paper in petri dishes for 10 days (10 h dark/14 h light; 15°C/20°C) and seedlings were transplanted to individual pots and raised in a climate chamber (10 h dark/14 h light; 15°C/20°C) subjected to different environmental treatments (see below).

### Experiment 1: Parental effects in AS34

Generation 1, parental stress exposure: AS34 plants were exposed to low nutrients, high salt concentration, jasmonic acid (JA) application, salicylic acid (SA) application, or control conditions (n = 8 plants per treatment), as described in Verhoeven et al. [25]. JA and SA are plant hormones involved in herbivore and pathogen signaling and defense reponses [42] and their application is often used to experimentally mimic biotic attack and to induce defense pathways. Nutrient and salt stresses were administered throughout the entire life of the plants by watering plants 1–2 per week with modified Hoagland solutions (compared to control: 5-fold dilution for nutrient stress and added NaCl to a 150 mM concentration for salt stress [25]). JA and SA (10 mM solutions) were applied to leaves twice during vegetative growth prior to the onset of flowering. In all treatments plants started flowering after 10–13 weeks and seeds were collected from each plant.

Generation 2, offspring trait evaluation: Offspring of control and stress-exposed parents were compared in different environments in four independent, completely randomized experiments (n = 8; each generation 1 plant contributed one offspring individual per experimental group). Effects of parental JA and SA exposure were evaluated in control environments. Effects of parental nutrient stress were evaluated in control and in low-nutrient environments. Effects of parental NaCl stress were evaluated in control and in high-NaCl environments. Experimental conditions were as described for generation 1. Before germination, the individual seed weight of each generation 2 plant was determined, in order to statistically account for maternal seed provisioning effects that can cause phenotypic differences among generation 2 plants. Plants were harvested 39 days (nutrient experiment), 44 days (salt experiment) or 59 days (JA and SA experiments) after transplanting. We recorded leaf length of a standardized fully extended leaf in the rosette, shoot dry biomass (after 48 h drying at 70°C) and specific leaf area (SLA; leaf area divided by leaf dry biomass, calculated based on four 8 mm diameter leaf discs taken from the distal part of two standardized fully extended leaves during harvesting). In the nutrient and salt experiments root dry biomass was determined in order to calculate root ∶ shoot biomass ratios. Dried shoots were ground and tissue P concentration (mg kg^−1^) were determined after H_2_SO_4_-salicylic acid-selenium digestion using colorimetry [43]. Several additional variables were measured (leaf dry matter content, leaf number, tissue K and Na content and tissue C∶N ratio) which were excluded from the analysis because they showed consistently high correlations with other measured variables (correlation coefficient r>0.7 in most cases) and were therefore considered to provide little added information.

Generation 2, caterpillar choice assay: 8-week old offspring of JA-exposed plants (JA-offspring) and offspring of control plants (control-offspring) were offered to larvae of the generalist herbivore *Spodoptera exigua* (beet armyworm; obtained from a lab culture) to test for parental effects of JA-treatment on herbivore feeding preference. One caterpillar (3^rd^ or 4^th^ instar) was put in the center of a petri dish on moist filter paper on which a circle of alternating leaf discs was placed from one JA-offspring plant (four discs) and one control-offspring plant (four discs; see Figure 3). After 30 minutes we determined visually whether caterpillars had eaten more from JA-offspring or from control-offspring. Each of 8 JA-offspring individuals was tested against three of the control-offspring individuals (24 independent tests in total).

### Experiment 2: Replication of parental effects in different apomictic genotypes

Generation 1, parental stress exposure: Genotypes AS34, A68 and H72 were exposed to control, JA-treatment, and low-nutrient conditions (10 plants per genotype per treatment) following the same procedures as described for experiment 1. JA was applied to leaves (10 mM, 0.75 ml) during vegetative growth eight weeks after transplanting.

Generation 2, offspring trait evaluation: Offspring of control and low-nutrient generation 1-plants were grown in control and low-nutrient environments (n = 10; each parent contributed one offspring individual per experimental group; nutrient levels as in experiment 1). The experiment followed a complete randomized block design with 10 blocks that were harvested consecutively 35–45 days after transplanting. In the statistical analysis, replicate blocks (and interactions with genotype and nutrient treatment) accounted for ontogenetic shifts in the measured traits during the 10-day period of harvesting. We measured root and shoot dry biomass and determined length, surface area (using WinFOLIA leaf scanning software, Regent Instruments) and dry biomass of the longest leaf. SLA was calculated on a total-leaf basis. Offspring of JA-treated and control plants were compared in a common control environment in a similar 10-block experimental design (n = 10), in which length, surface area and dry biomass of the longest leaf were determined 35–45 days after transplanting. Additional plants were grown in a common control environment to test *S. exigua* feeding preference as described for experiment 1, using leaf discs from 5-week old plants and testing each JA-offspring individual three times against a control-offspring individual (30 feeding trials per apomictic genotype).

### Experiment 3: zebularine effects on nutrient stress response

After exposing experiment 2-plants for two consecutive generations to either low-nutrient or control environments, seeds ( = generation 3) from 10 AS34 plants per environment were pooled, surface-sterilized in a 0.5% sodium hypochlorite solution (15 min) and germinated in petri dishes on solidified 0.8% agar containing 0, 10, 25, 50 or 100 µM zebularine (Sigma-Aldrich). Zebularine is known to cause transient and partial DNA-demethylation in a way that is more indiscriminant to sequence context than 5-azacytidine [44]. After 14 days seedlings were transplanted to pots containing a nutrient-poor sand/potting soil mixture and grown at control and low-nutrient conditions following the same procedures and experimental design as described for experiment 2 (2 parental nutrient environments×2 offspring nutrient environments×5 zebularine germination levels×10 complete randomized replicate blocks = 200 plants in total). Plants were harvested by block 45–47 days after transplanting. We measured root and shoot dry biomass; length, surface area and dry biomass of the longest leaf; and total leaf area of all leaves combined. Many plants failed to grow or died in the 50 and 100 µM zebularine treatments and the analysis is restricted to the 0, 10 and 25 µM zebularine treatments (with 6–10 surviving plants per experimental group).

In all three experiments fixed-effect ANOVA models were fitted that accounted for block effects and for individual differences in initial seed weight (in experiments 1 and 2), using SAS for Windows version 9.2 (The SAS Institute, Cary, NC).
